# Patterns of Antibiotic Prescribing for Pediatric Outpatient Visits in the United States: Findings From the National Ambulatory Medical Care Survey 2010-2015

**DOI:** 10.7759/cureus.98245

**Published:** 2025-12-01

**Authors:** Okelue E Okobi, Emmanuel Olawusi, Wuraola R Awosan, Chibuzo N Nwodo, Ifesinachi Nwankwor, Angelina Uzor

**Affiliations:** 1 Family Medicine, Larkin Community Hospital Palm Springs Campus, Miami, USA; 2 Family Medicine, IMG Research Academy and Consulting LLC, Homestead, USA; 3 Pediatrics, Maimonides Medical Center, New York City, USA; 4 Medicine, Ternopil National Medical University, Ternopil, UKR; 5 Ear, Nose, and Throat (ENT), Royal Derby Hospital, Derby, GBR; 6 General Practice, General Hospital Agulu, Agulu, NGA; 7 Public Health, East Tennessee State University, Johnson City, USA

**Keywords:** antibiotic stewardship, antimicrobial resistance, namcs, outpatient care, pediatrics, prescribing patterns

## Abstract

Background: Inappropriate antibiotic prescribing for children remains a major public health concern, contributing to antimicrobial resistance (AMR), adverse events, and excess healthcare costs. This study examined prescribing patterns in United States (US) pediatric outpatient visits between 2010 and 2015 using the National Ambulatory Medical Care Survey (NAMCS).

Methods: We conducted a cross-sectional analysis of outpatient encounters for patients aged 0-17 years. Antibiotic prescribing was evaluated by diagnosis category, age group, and patient characteristics. Descriptive statistics and adjusted probabilities were generated. Missing data were assessed, with no imputation applied, as most key variables were complete.

Results: Among 45,666 unweighted pediatric visits, representing 991,561,251 weighted visits, the strongest predictor of antibiotic use was the type of diagnosis. Bacterial infections, particularly otitis media and urinary tract infections, had the highest prescribing probabilities, while non-infectious diagnoses rarely received antibiotics. Respiratory illnesses accounted for substantial prescribing, consistent with prior reports of inappropriate use.

Conclusion: Pediatric antibiotic prescribing during 2010-2015 was primarily diagnosis-driven, with persistent overuse for respiratory conditions. These results underscore the need for strengthened outpatient stewardship strategies to reduce inappropriate use and support guideline-concordant care.

## Introduction

In the United States (US) and the world, prescribing antibiotics to pediatric patients continues to be a serious public health concern [1]. Although antibiotics are vital for treating various bacterial infections, their inappropriate or unnecessary use significantly contributes to the emergence of antimicrobial resistance (AMR), adverse drug events, and rising health expenditures, according to the Centers for Disease Control and Prevention (CDC) [2,3]. The World Health Organization (WHO) defines AMR as one of the greatest health risks all over the globe, and children are especially vulnerable because they have a lot of contact with healthcare specialists and their immune system is not fully developed [4]. In the US, outpatient settings, especially primary care and urgent care clinics, account for a majority of antibiotic prescriptions, making them a focal point for antibiotic stewardship efforts [5].

Prescribing patterns also differ based on age group, category of the diagnosis, and geography [6]. Younger children, including children below five years, tend to receive more antibiotic prescriptions in part because there are more cases of acute otitis media and fears of complications by clinicians [7,8]. Variation in regionality was observed in national data, and consistently, the South had higher prescribing rates compared to the West, which could be an indication of some neurologic practice differences, patient expectations, and healthcare accessibility [9].

Past studies have reported that a significant number of pediatric antibiotic prescriptions are made in instances in which antibiotics are not required, such as upper respiratory tract infections that are not viral [10]. It is estimated that up to 30% of outpatient prescriptions of antibiotics in children in the US are inappropriate, and, as such, they pose a challenge to achieving guideline-concordant prescriptions [11]. Respiratory diagnoses are frequently subject to improper use, and these diagnoses represent the highest cause of outpatient visits in pediatrics.

A substantial period that is worth exploring is the experience from 2010 to 2015 concerning trends in pediatric outpatient prescribing in the US [12]. During this time, various national and institutional efforts to enhance antibiotic stewardship were observed, such as the CDC’s Get Smart effort and publication of revised clinical guidelines on common pediatric infections [13]. Technology levies were also observed during this period, such as increased use of electronic health records and other clinical decision support systems, which may influence prescribing patterns as well [14].

The National Ambulatory Medical Care Survey (NAMCS) is a unique nationally representative dataset that is used to study prescribing trends in outpatient care [15,16]. Since its establishment, NAMCS has played a critical role in the surveillance of healthcare usage patterns, including medication prescribing by clinicians across various patient demographic groups and clinical situations [17]. Pediatric outpatient visits offer a specific possibility to evaluate the relevance of antibiotic utilization and the necessity of antibiotic prescription [18,19]. Outpatient care is also very different than inpatient care; patients have shorter visits, more turnover, and in some cases less diagnostic testing, all of which may promote antibiotic dispensation empirically even when viral causes are more probable [20]. Using the NAMCS public use database between 2010 and 2015, the research aimed to define the percentage of pediatric outpatient encounters (ages 0 to 17 years) leading to an antibiotic prescription and investigate the differences depending on the type of diagnosis, patient age, and US region.

## Materials and methods

Study design and data source

This cross-sectional study utilized data from the NAMCS public use files for the years 2010-2015 [21]. NAMCS is an annual, nationally representative survey conducted by the National Center for Health Statistics (NCHS) that employs a multistage probability sampling design to collect data on visits to non-federally employed, office-based physicians in the US. The survey captures detailed patient, provider, and visit characteristics, including demographic information, diagnoses coded according to the International Classification of Diseases, Clinical Modification (ICD-CM), and medications prescribed during the visit [22]. For the purpose of this analysis, we restricted the dataset to pediatric visits, defined as patients aged 0-17 years, excluding prenatal and maternal care encounters.

Study population and inclusion criteria

We included all outpatient visits made by patients aged 0-17 years during the 2010-2015 survey years, in which diagnoses were recorded exclusively in the International Classification of Diseases, Ninth Revision, Clinical Modification (ICD-9-CM) [22]. Visits were excluded if age was missing or if the patient was older than 17 years. Encounters were also excluded if the visit was classified as a follow-up for an ongoing antibiotic prescription without a new diagnostic assessment, to avoid overcounting antibiotic use for the same episode of illness.

Measures

The primary outcome was whether an antibiotic was prescribed during the visit. NAMCS records up to 30 medications per encounter (RX1-RX30) with associated MULTUM therapeutic class codes (Multum Lexicon Therapeutic Classification). A visit was coded as antibiotic-positive if any medication had MULTUM Level 1 = “001” (antimicrobials) and Level 2 = “009, 011, 012, 013” (penicillins and related antibacterials, macrolides/lincosamides, cephalosporins, or other antibacterials). This definition captures systemic antibacterials, including penicillins, cephalosporins, and macrolides, while excluding antivirals, antifungals, and antiparasitics.

To link prescribing to clinical indications, we classified visits using the primary diagnosis recode (DIAG1R). Because our objective was to focus on conditions most relevant to outpatient antibiotic stewardship in children, we defined mutually exclusive diagnosis groups centered on common pediatric infections, with acute respiratory infections deliberately restricted to the ICD-9-CM 460-466 block rather than the entire respiratory chapter (460-519) to isolate conditions where antibiotics are frequently unnecessary or guideline-discordant, thereby aligning the exposure with the stewardship question. The exact DIAG1R ranges are shown in Table 1.

**Table 1:** Classification of primary diagnoses (ICD-9-CM via DIAG1R) used for analysis. DIAG1R (primary diagnosis recode) is derived from NAMCS 2010-2015 [21]. ICD-9-CM codes are sourced from ICD-CM [22]. NAMCS and ICD-CM [21,22] are publicly available datasets. ICD-CM: International Classification of Diseases, Clinical Modification; ICD-9-CM: International Classification of Diseases, Ninth Revision, Clinical Modification

Diagnosis category	ICD-9-CM codes	DIAG1R recode range	Conditions
Acute respiratory infections	460-466	146000-146699	Common cold, acute sinusitis, acute pharyngitis/tonsillitis, laryngitis, acute bronchitis/bronchiolitis
Otitis media	381-382	138100-138299	Acute non-suppurative otitis media, suppurative otitis media
Skin and soft tissue infections	680-686	168000-168699	Cellulitis, abscess, impetigo, other local skin infections
Urinary tract infections (unspecified site)	599.0	159900	Urinary tract infection, site not specified
Other specific bacterial infections	034-035	103400-103599	Streptococcal sore throat, scarlet fever
Non-infectious/other diagnoses	All other ICD-9-CM codes	All other DIAG1R values	Injuries, chronic diseases, preventive care, non-infectious encounters

For the covariates, age was categorized as 0-1, 2-5, 6-12, and 13-17 years to reflect clinically meaningful pediatric strata. Additional covariates included patient sex and race/ethnicity. These variables were selected a priori based on prior literature showing age-related variation in antibiotic prescribing and because they are consistently available across NAMCS years.

Data completeness

Prior to conducting the analyses, we assessed the extent of missing data across all study variables. The core analytic variables age group, sex, race/ethnicity, diagnosis category, and antibiotic prescription status had no missing observations, allowing all analyses to be performed using complete-case data without the need for imputation. In evaluating data availability, we identified that the geographic region variable was missing for 76.6% of visits. Because this level of missingness would compromise the validity of descriptive comparisons and multivariable modeling, the geographic region variable was excluded from all analyses. This approach ensured that the regression model and descriptive findings were based solely on variables with complete information, thereby improving the reliability and interpretability of the results. The absence of regional data is acknowledged as a limitation of the study.

Statistical analyses

All analyses were performed using STATA version 18 (StataCorp LLC, College Station, US), accounting for the complex survey design, visit weights, and clustering to produce nationally representative estimates. Descriptive statistics were calculated to summarize patient demographics, diagnosis categories, and antibiotic prescribing frequencies. The proportion of visits resulting in an antibiotic prescription was estimated overall and stratified by diagnosis category, patient age group, and region. Differences between groups were assessed using a design F-test for all categorical variables. Multivariable logistic regression models were constructed to evaluate independent associations between patient/visit characteristics and the likelihood of antibiotic prescribing, with results reported as adjusted ORs and 95% CIs. Statistical significance was set at a two-sided p-value of <0.05.

To evaluate collinearity among predictors, variance inflation factors (VIFs) were calculated. All covariates demonstrated VIF values between 1.00 and 1.54, with a mean VIF of 1.14, indicating low correlation between independent variables. Given that these values fall well below conventional cutoffs for concern, multicollinearity was not considered problematic, and all covariates were retained in the regression models.

Ethical considerations

This study was exempt from institutional review board (IRB) oversight as it utilized publicly available, de-identified NAMCS data provided by the NCHS. No individual patient identifiers were accessed, and all analyses were conducted in compliance with the ethical standards outlined in the Declaration of Helsinki and relevant US federal regulations regarding secondary data use.

## Results

Table 2 presents the distribution of antibiotic prescribing across demographic and clinical characteristics among pediatric outpatient visits in the US between 2010 and 2015. The analysis included an estimated 991,561,251 weighted visits, of which 173,269,592 (17%) resulted in an antibiotic prescription. Significant variation in prescribing was observed across diagnosis categories and age groups, while patterns by sex, race/ethnicity, and geographic region were less pronounced.

**Table 2:** Distribution of patient and visit characteristics by antibiotic prescription status among US pediatric outpatient visits, NAMCS 2010-2015. Values represent weighted frequencies and proportions of pediatric outpatient visits with or without an antibiotic prescription. Statistical comparisons are based on survey-weighted, design-based F-tests for categorical variables. Diagnosis categories are based on the ICD-9-CM coding structure [22]. NAMCS and ICD-CM [21,22] are publicly available datasets. NAMCS: National Ambulatory Medical Care Survey; ICD-CM: International Classification of Diseases, Clinical Modification; ICD-9-CM: International Classification of Diseases, Ninth Revision, Clinical Modification; US: United States

Characteristic	Weighted visits (N=991,561,251)	No antibiotic (N=818,291,658)	Antibiotic (N=173,269,592)	F-test	p-value
Patient gender (n, %)	-	-	-	2.98	0.084
Male	517,107,284	429,062,569 (83%)	88,044,715 (17%)	-	-
Female	474,453,966	389,229,089 (82%)	85,224,877 (18%)	-	-
Diagnosis category (n, %)	-	-	-	659.06	<0.001
Acute respiratory infection	103,800,206	65,416,724 (63%)	38,383,481 (37%)	-	-
Otitis media	51,347,440	14,752,335 (29%)	36,595,105 (71%)	-	-
Skin/soft tissue infection	8,672,880	4,269,294 (49%)	4,403,586(51%)	-	-
Urinary tract infection	3,352,103	1,254,377 (37%)	2,097,725 (63%)	-	-
Other bacterial infection	9,708,182	1,057,815 (11%)	8,650,366 (89%)	-	-
Non-infectious	814,680,437	731,541,110 (90%)	83,139,326 (10%)	-	-
Race/ethnicity (n, %)	-	-	-	2.67	0.048
Non-Hispanic White	611,483,263	502,754,017 (82%)	108,729,246 (18%)	-	-
Non-Hispanic Black	103,680,639	86,746,298 (84%)	16,934,341 (16%)	-	-
Hispanic	212,022,954	173,447,044 (82%)	38,575,909 (18%)	-	-
Non-Hispanic Other	64,374,393	55,344,298 (86%)	9,030,095 (14%)	-	-
Age group (in years) (n, %)	-	-	-	63.1	<0.001
0-1	255,597,872	218,436,333 (85%)	37,161,539 (15%)	-	-
2-5	221,044,127	167,634,812 (76%)	53,409,315 (24%)	-	-
6-12	290,318,489	235,995,268 (81%)	54,323,221 (19%)	-	-
13-17	224,600,761	196,225,244 (87%)	28,375,516 (13%)	-	-

Across all visits, antibiotic prescribing did not differ significantly by patient sex (p=0.084). Among male patients, 88,044,715 (17%) visits included an antibiotic prescription compared to 429,062,569 (83%) visits without. Similarly, among female patients, 85,224,877 (18%) visits involved an antibiotic versus 389,229,089 (82%) without.

The diagnosis category was strongly associated with antibiotic prescribing (p<0.001). Children diagnosed with acute respiratory infections accounted for 103,800,206 visits, of which 38,383,481 (37%) involved antibiotics, and 65,416,724 (63%) did not. Otitis media showed the highest relative prescribing, with 36,595,105 (71%) visits receiving an antibiotic compared to 14,752,335 (29%) without. Antibiotics were also prescribed in 4,403,586 skin/soft tissue infection visits (51%) and 2,097,725 urinary tract infection visits (63%). Other specific bacterial infections were overwhelmingly treated with antibiotics, with 8,650,366 visits (89%) compared to only 1,057,815 (11%) managed without. In contrast, non-infectious encounters had a low prescribing rate, with only 83,139,326 (10%) visits involving antibiotics out of 814,680,437 total.

Differences by race/ethnicity reached statistical significance (p=0.048) but were relatively modest in magnitude. Among non-Hispanic White patients (n=611,483,263), antibiotics were prescribed in 108,729,246 (18%) visits. Non-Hispanic Black patients (n=103,680,639) had the lowest proportion at 16,934,341 (16%). Hispanic patients (n=212,022,954) had 38,575,909 visits (18%) with antibiotics, while non-Hispanic patients of other races (n=64,374,393) had the lowest relative proportion at 9,030,095 (14%).

Age group was also significantly associated with prescribing (p<0.001). Infants aged 0-1 year accounted for 255,597,872 visits, with 37,161,539 (15%) involving antibiotics. Children aged 2-5 years (n=221,044,127) had a higher proportion, with 53,409,315 (24%) visits including antibiotics. For school-aged children 6-12 years (n=290,318,489), antibiotics were prescribed in 54,323,221 visits (19%), while adolescents 13-17 years (n=224,600,761) had the lowest relative prescribing at 28,375,516 visits (13%).

Table 3 presents the results of the multivariable logistic regression model evaluating predictors of antibiotic prescribing during pediatric outpatient visits. The analysis adjusted for demographic and clinical characteristics, with non-infectious diagnoses and infants aged 0-1 year serving as the reference categories. Several strong and statistically significant associations were observed, particularly across diagnosis groups and younger age strata.

**Table 3:** Multivariable logistic regression of factors associated with antibiotic prescribing among US pediatric outpatient visits, NAMCS 2010-2015. ORs with 95% CIs are reported for the likelihood of receiving an antibiotic prescription compared with the reference group. Reference categories include: non-infectious diagnoses for diagnosis category; 0-1 year for age group; male sex; non-Hispanic White race/ethnicity. Diagnosis categories are based on the ICD-9-CM coding structure [22]. NAMCS and ICD-CM [21,22] are publicly available datasets. NAMCS: National Ambulatory Medical Care Survey; ICD-CM: International Classification of Diseases, Clinical Modification; ICD-9-CM: International Classification of Diseases, Ninth Revision, Clinical Modification; US: United States

Predictor	OR	95% CI	p-value
Female	1.06	(0.98-1.16)	0.160
Age group (in years)	-	-	-
2-5	1.74	(1.52-2.01)	<0.001
6-12	1.55	(1.35-1.77)	<0.001
13-17	1.17	(1.02-1.34)	0.023
Diagnosis category	-	-	-
Acute respiratory infection	5.05	(4.42-5.76)	<0.001
Otitis media	21.87	(18.26-26.19)	<0.001
Skin/soft tissue infection	9.02	(6.57-12.38)	<0.001
Urinary tract infection	13.86	(9.12-21.08)	<0.001
Other bacterial infection	65.01	(39.45-107.15)	<0.001
Race/ethnicity	-	-	-
Non-Hispanic Black	1.00	(0.83-1.22)	0.968
Hispanic	1.11	(0.97-1.28)	0.126
Non-Hispanic Other	0.84	(0.67-1.06)	0.143

Sex was not significantly associated with antibiotic prescribing, although female patients had slightly higher odds compared to male patients (OR=1.06, 95% CI: 0.98-1.16, p=0.160).

Age group demonstrated notable variation. Compared to infants aged 0-1 year, children aged 2-5 years had 1.74 times higher odds of receiving an antibiotic (95% CI: 1.52-2.01, p<0.001). School-aged children 6-12 years also had significantly higher odds (OR=1.55, 95% CI: 1.35-1.77, p<0.001). Adolescents 13-17 years showed a modest, significant increase (OR=1.17, 95% CI: 1.02-1.34, p=0.023).

The diagnosis category was the strongest predictor of antibiotic prescribing. Relative to non-infectious diagnoses, acute respiratory infections were associated with 5.05 times higher odds of antibiotic prescribing (95% CI: 4.42-5.76, p<0.001). Otitis media showed the highest magnitude of association, with children being 21.87 times more likely to receive an antibiotic (95% CI: 18.26-26.19, p<0.001). Other significant predictors included skin and soft tissue infections (OR=9.02, 95% CI: 6.57-12.38, p<0.001), urinary tract infections (OR=13.86, 95% CI: 9.12-21.08, p<0.001), and other specific bacterial infections (OR=65.01, 95% CI: 39.45-107.15, p<0.001). 

Race and ethnicity were not significantly associated with prescribing. Compared to non-Hispanic White patients, non-Hispanic Black patients (OR=1.00, 95% CI: 0.83-1.22, p=0.968), Hispanic patients (OR=1.11, 95% CI: 0.97-1.28, p=0.126), and non-Hispanic patients of other races (OR=0.84, 95% CI: 0.67-1.06, p=0.143) showed no statistically significant differences.

Taken together, the model highlights diagnosis category as the strongest determinant of antibiotic prescribing in pediatric outpatient visits, with age group also contributing significantly, while sex and race/ethnicity did not exhibit independent associations.

**Figure 1:**
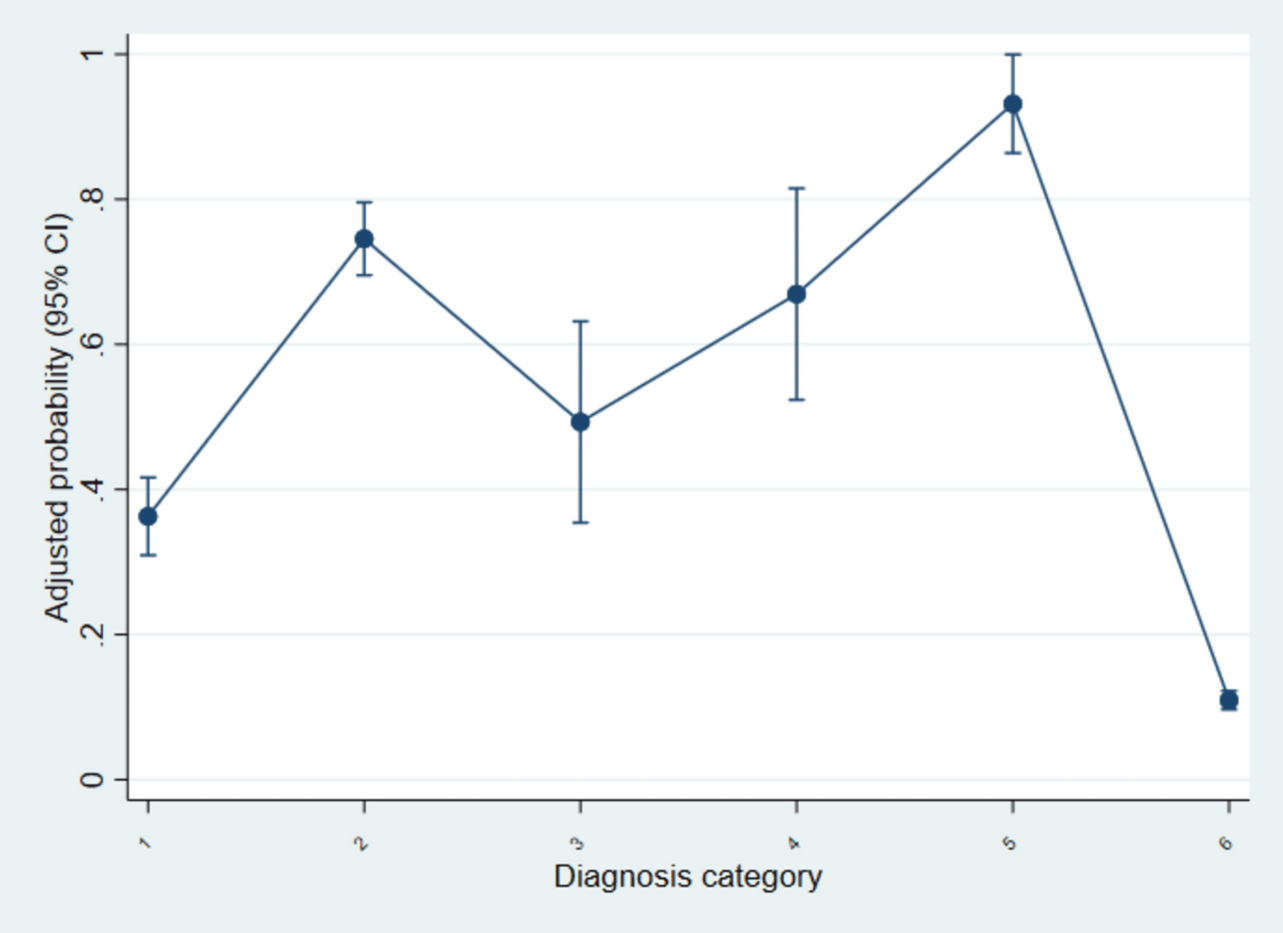
Adjusted probability of antibiotic prescribing by diagnosis category among US pediatric outpatient visits, NAMCS 2010-2015. Predicted probabilities of antibiotic prescribing with 95% CIs are shown by diagnosis category, derived from the multivariable logistic regression model. The categories correspond to: (1) acute respiratory infections, (2) otitis media, (3) skin and soft tissue infections, (4) urinary tract infections, (5) other bacterial infections, and (6) non-infectious conditions (reference). Diagnosis categories are based on the ICD-9-CM coding structure [22]. NAMCS and ICD-CM [21,22] are publicly available datasets. NAMCS: National Ambulatory Medical Care Survey; ICD-CM: International Classification of Diseases, Clinical Modification; ICD-9-CM: International Classification of Diseases, Ninth Revision, Clinical Modification; US: United States

Figure 1 illustrates the adjusted probability of antibiotic prescribing across diagnosis categories after controlling for demographic and regional factors. The plot highlights substantial variation in the likelihood of antibiotic use, with the highest probabilities observed for bacterial infections and otitis media, and the lowest for non-infectious conditions.

The adjusted probability of antibiotic prescribing varied markedly by diagnosis category. Among acute respiratory infections (category 1), the predicted probability was approximately 0.35, increasing sharply for otitis media (category 2) to around 0.75. Skin and soft tissue infections (category 3) showed a moderate probability near 0.50, while urinary tract infections (category 4) had a higher predicted probability close to 0.67. Other bacterial infections (category 5) displayed the highest probability of prescribing, approaching 0.90. In contrast, non-infectious conditions (category 6) had a markedly lower probability of antibiotic use, estimated at just above 0.10. These results visually reinforce the regression findings in Table 3, underscoring diagnosis type as the dominant driver of prescribing behavior in pediatric outpatient visits.

## Discussion

This study examined antibiotic prescribing patterns in US pediatric outpatient visits between 2010 and 2015 using the nationally representative NAMCS database. The results show that diagnosis type was the strongest determinant of prescribing, with markedly higher probabilities for otitis media, urinary tract infections, and other bacterial conditions, while non-infectious diagnoses were rarely treated with antibiotics. These findings align with previous reports indicating that diagnostic category is the single most important driver of prescribing decisions in pediatric outpatient care, particularly where diagnostic testing is limited and empirical prescribing is common [6-8,10].

Even though most causes of upper respiratory tract infections are viral in origin, with rhinovirus constituting 50-80% of the viral cases, most cases end up being treated with antibiotics [23,24]. Parent and caregiver expectations play a significant role in shaping antibiotic prescribing practices in pediatric outpatient settings, and pressures from parents have been noted as one of the factors that influence antibiotic prescriptions [25]. A systematic review showed that over 50% of parents expect antibiotic prescriptions for the treatment of upper respiratory tract infection in their children [26]. This can potentially lead to AMR and therapeutic failure in the future [26]. Furthermore, children will be unnecessarily exposed to potential side effects attributed to the individual antibiotics.

This attitude of parents to antibiotics is influenced by poor understanding of antibiotic use and also partly by previous experiences [27]. Therefore, despite the pressure and expectation from parents, clinicians should strive to avoid injudicious prescription of antibiotics. Shared decision-making and educating parents on the possible risks associated with inappropriate use of antibiotics could help address this [24,26].

The high adjusted probability of prescribing for otitis media mirrors earlier studies showing that children, especially those under five years, are frequent recipients of antibiotics, sometimes beyond guideline recommendations [7,11]. While antibiotic treatment is warranted for many bacterial infections, prior research has consistently highlighted overuse for viral respiratory illnesses [10,15]. Our results reinforce these concerns, as a notable proportion of respiratory visits still resulted in antibiotic prescriptions, echoing national estimates that nearly one-third of outpatient prescriptions in children are unnecessary [3,11].

Geographic variation in prescribing has been well documented, with the South showing higher rates compared to other regions [9]. However, in our study, over three-quarters of encounters lacked geographic data, which limited our ability to evaluate regional trends. Despite this, the findings highlight how diagnostic drivers persist as the central influence on antibiotic use.

The study period of 2010-2015 represents an important transitional phase in US outpatient pediatric care, as it coincided with growing national efforts to improve antibiotic stewardship, including the CDC’s Get Smart campaign and updates to pediatric prescribing guidelines [13]. Because these initiatives were still gaining momentum, the prescribing patterns observed in our analysis provide a meaningful historical baseline for understanding how outpatient pediatric antibiotic use has evolved in subsequent years [11,15]. After 2015, stewardship activities became more widespread, supported by broader implementation of antimicrobial stewardship programs, increased reliance on electronic decision-support tools, and heightened national attention to AMR [2,14]. Additionally, the COVID-19 pandemic fundamentally changed respiratory illness epidemiology and outpatient prescribing behaviors, which makes pre-pandemic prescribing patterns such as those captured in our dataset especially valuable for comparison. Taken together, these factors emphasize the continued relevance of our findings for understanding the long-term influence of early stewardship initiatives and for identifying areas where inappropriate prescribing persists despite ongoing national efforts.

Strengths and limitations

A major strength of this study is the use of NAMCS, a nationally representative dataset that captures outpatient care across diverse populations and clinical contexts. Its large sample size and standardized data collection strengthen the reliability of our findings.

Nonetheless, several limitations must be noted. First, the high proportion of missing data on geographic region (76.6%) restricted our ability to evaluate regional variation, which is an important factor in prescribing behavior, and therefore led to the exclusion of this variable from the analysis. Second, the visit-level structure of NAMCS may misclassify diagnoses or fail to capture the clinical nuances behind prescribing decisions. Third, the dataset records prescriptions issued but not whether they were filled, meaning actual antibiotic consumption cannot be confirmed. Finally, as a cross-sectional survey, NAMCS does not allow causal inferences about stewardship interventions or changes over time.

From the findings in our study and supporting evidence, we recommended that pediatric clinics should adopt the CDC outpatient antibiotic stewardship intervention, which addresses clinician monitoring and education to ensure the guideline is adhered to [13,20]. Furthermore, we recommend that children under five and patients with otitis media or acute respiratory infection are the groups at higher risk of getting unnecessary antibiotic prescriptions. Strategies like providing a diagnostic and prescription guideline should be put in place to curb antibiotic overuse in these groups [7,10,11].

## Conclusions

Antibiotic prescribing in US pediatric outpatient settings between 2010 and 2015 was mainly driven by clinical diagnosis, with the greatest use observed for bacterial infections such as otitis media and urinary tract infections. Despite national stewardship initiatives introduced during this period, antibiotics continued to be prescribed for many respiratory conditions where they were likely unnecessary, indicating that further progress is still needed. These results highlight that diagnosis type remains the strongest influence on prescribing decisions, but also point to meaningful opportunities to bring daily clinical practice closer to evidence-based guidelines.

Ongoing focused efforts in outpatient antibiotic stewardship are essential to limit overuse and reduce the growing threat of AMR among children. Future strategies should strengthen clinician support systems, expand education for healthcare providers, and engage parents to correct common misconceptions about when antibiotics are truly needed. Continued monitoring through national programs such as NAMCS will be vital to track improvements and guide data-informed interventions that promote safer, more responsible antibiotic use in pediatric care.
